# Generation of a selectively cytotoxic fusion protein against p53 mutated cancers

**DOI:** 10.1186/1471-2407-12-338

**Published:** 2012-08-03

**Authors:** Christina A Kousparou, Efthymia Yiacoumi, Mahendra P Deonarain, Agamemnon A Epenetos

**Affiliations:** 1 Trojantec Ltd, The Bank of Cyprus Oncology Centre, 32 Acropoleos Avenue, 2006, Nicosia, Cyprus; 2 Imperial College London, Exhibition Road, London, SW7 2AZ, UK

**Keywords:** Cancer therapy, Antennapedia, p21, Trojan, Recombinant fusion protein, Cell-penetrating

## Abstract

**Background:**

A significant number of cancers are caused by defects in p21 causing functional defects in p21 or p53 tumour-suppressor proteins. This has led to many therapeutic approaches including restoration by gene therapy with wild-type p53 or p21 using viral or liposomal vectors, which have toxicity or side-effect limitations. We set out to develop a safer, novel fusion protein which has the ability to reconstitute cancer cell lines with active p21 by protein transduction.

**Methods:**

The fusion protein was produced from the cell-translocating peptide *Antennapedia* (Antp) and wild-type, full-length p21 (Antp-p21). This was expressed and refolded from *E. coli* and tested on a variety of cell lines and tumours (in a BALB/c nude xenograft model) with differing p21 or p53 status.

**Results:**

Antp-p21 penetrated and killed cancer cells that do not express wild type p53 or p21. This included cells that were matched to cogenic parental cell lines. Antp-p21 killed cancer cells selectively that were malignant as a result of mutations or nuclear exclusion of the p53 and p21 genes and over-expression of MDM2. Non-specific toxicity was excluded by showing that Antp-p21 penetrated but did not kill p53- or p21- wild-type cells. Antp-p21 was not immunogenic in normal New Zealand White rabbits. Recombinant Antp peptide alone was not cytotoxic, showing that killing was due to the transduction of the p21 component of Antp-p21. Antp-p21 was shown to penetrate cancer cells engrafted *in vivo* and resulted in tumour eradication when administered with conventionally-used chemotherapeutic agents, which alone were unable to produce such an effect.

**Conclusions:**

Antp-p21 may represent a new and promising targeted therapy for patients with p53-associated cancers supporting the concept that rational design of therapies directed against specific cancer mutations will play a part in the future of medical oncology.

## Background

Uncontrolled cellular proliferation and tumour development can result from a malfunction in cell cycle control which is largely regulated by cyclins and cyclin-dependent kinases (CDKs) through phosphorylation. This is reflected by the fact that a large number of cancers involve mutations in the p53 and p21 tumour suppressor proteins [1], which are key components in the control of cyclin/CDK phosphorylation and complex formation. CDK inhibitor p21 (WAF1/CIp1/SDI1) plays a key role in cell cycle G1-arrest in response to DNA damage [2,3], and is involved in the assembly of active cyclin–kinase complexes [4,5]. Specifically, it blocks cycD/cdk4 complex formation, which, in turn, keeps the retinoblastoma (Rb) protein in a state of low phosphorylation and tightly bound to the transcription factor E2F, inhibiting its activity. It also inhibits proliferating cell nuclear antigen (PCNA)-dependent DNA replication [6]. In addition to cell-cycle control, p21 participates in important cell processes such as differentiation, senescence, apoptosis and it even has a possible role in homeostatic controls in adult stem cell processes [7] and cancer stem cells [8].

Reconstituting p53 in tumours has been accomplished with both retroviral and adenoviral vectors [9], resulting in significant growth inhibition [10,11]. Contusugene ladenovec (Advexin®) is a chimeric adenovirus which delivers functional p53 to head and neck tumours [12]. Preliminary results from advanced clinical trials were promising showing a significant increase in survival when patient p53-status was considered. Similarly, adenovirus-delivered p21 inhibited tumour growth causing cell cycle arrest at G0/G1 and altering tumour morphology [13,14]. However, there are still concerns and limitations with viral delivery of therapeutic genes [15,16], which have hampered their clinical and commercial development [17].

Other delivery systems, including liposomes can suffer from unstable formulations, poor pharmacokinetics, and scavenging by the immuno/reticulo-endothelial systems [18].

Peptide translocation across impermeable biological membranes through a receptor-independent mechanism [19-21], has unveiled novel possibilities in biopharmaceutical research. The so-called ‘Trojan horse’ peptides are regions within proteins, otherwise called protein-transduction domains (PTDs) or cell-penetrating peptides (CPPs), which have the ability to traverse biological membranes efficiently including the blood–brain barrier [22-24], in a temperature-, receptor- and transporter-independent fashion. They have therapeutic potential because they can transport any pharmaceutical compound [23,24]. Examples include the Tat peptide from the HIV-1 virus and the *drosophila* homeotic transcription factor Antp, the protein product of the antennapedia gene. The mechanism of transduction relies on the presence of a cationic helix which is important for lipid interactions and for penetration of the membrane [24].

Others and we reasoned that anti-mitotic therapy would be less effective if administered to a tumour which is defective in the proteins needed to cause G1 arrest/apoptosis. However, if wild-type tumour-suppressor protein function is restored by protein complementation, the cells will have regained drug sensitivity. Groups have used either truncated or synthetic peptides to identify regions of p21 that affect the cell-cycle [25-27]. Linking one of the three helices from *Antennapedia* to peptides corresponding to residues 17–33 or 63–77 of p21 inhibited growth of two human ovarian cancer cell lines [25]. Similarly, linkage to residues 141–160 of p21 decreased cells in S phase and blocked phosphorylation of Rb *in vivo*[27]. However, in previous studies, activity was impaired because truncated versions of *Antennapedia* could only deliver truncated versions of p21. Additionally, the small size of these peptides made pharmacological development of this approach unfeasible due to rapid systemic clearance. The multi-functional role and interactions of p21 suggests its presence in a cell is required in an intact form [7].

In the experiments presented herein, we describe a full-length Antp-p21 fusion protein in order to overcome the obstacle of cell membrane penetration. We demonstrate that the fusion protein accumulates in cells *in vitro* and *in vivo* resulting in cell division arrest and apoptosis, leading to tumour growth inhibition and prolonged survival, an effect enhanced by anti-mitotic chemotherapy. Subcutaneous human tumour xenografts implanted into immuno-compromised mice are a well-established system for studying cancer drug behaviour allowing drug delivery in the context of living tissues and the observation of therapeutic and side effects. The significance of these results is discussed in the context of future cancer therapeutics.

## Methods

### Chemicals and antibodies

Chemicals were from Sigma, Fluka & VWR. Chromatography media and general immunochemicals from GE Healthcare, UK. Anti-p21WAF1 mouse monoclonal IgG was from Oncogene and anti-pentahistidine antibody was from Qiagen.

### Cell lines

Cancer cell lines were obtained from the American Type Tissue Collection (Rockville, MD) and included SKOV3 human ovarian cancer cells that do not express p53, RKO and RKO-E6 human colon carcinoma cells which contain a stably integrated human papilloma virus (HPV) E6 oncogene which causes a decrease in normal p53 levels and functions, including the transactivation of p21. Colo-205 colorectal cancer cells express wild-type p21 and p53. A panel of HCT116 cogenic human colorectal cancer cell lines with different p53 and p21 statuses (p53^+^/p21^+^, p53^-^/p21^+^, p21^-^/p53^+^, p21^-^/p53^-^) was the kind gift of Prof. B. Vogelstein (John Hopkins University, USA) [28].

### Cloning, expression, and purification of Antp, p21 and Antp-p21

The p21 gene was provided from pcDNA3-p21 (Dr Mann, Imperial College) and cloned in vector pDS56 (Novagen). This construct was used to express recombinant p21, with an N-terminal polyhistidine tag for purification (pDS56p21). The Antennapedia homeodomain (Antp) gene was subcloned from pA4C1 (Prof. Crisanti, Imperial College) into vector pDS56 for bacterial expression (pDS56Antp). The fusion protein was expressed from a construct (pDS56Antp-p21) by subcloning Antp directly upstream of p21. DNA Constructs were transformed into the *E. coli* M15 and selected on 2TYAmp/Kan plates. The Antp and Antp-p21 proteins were expressed as insoluble inclusion bodies and refolded by denaturation and serial dialysis as described in the supplementary methods.

### Characterization of recombinant Antp-p21

Proteins were identified by SDS-PAGE and immunoblotting with anti-p21WAF1 mouse monoclonal IgG or anti-pentahistidine antibody followed by peroxidase-conjugated antibodies. Samples containing pure proteins were pooled and dialysed against Tris-buffered saline (20 mM Tris–HCl, pH 7.5, 0.5 M NaCl, 0.1% Tween-20) and then two changes of PBS (150 mM potassium phosphate, 0.7% NaCl, pH 7.4) for 16 hours at 4°C. The dialysed proteins were concentrated by ultrafiltration to 2.5 mg/ml.

Penetration of recombinant Antp and Antp-p21 was measured by incubating 100 μg/ml proteins with SKOV3 cells in serum-free media. The cells were washed with PBS containing Tween-20, fixed with 4% paraformaldehyde and stained with 1:100 anti-pentahistidine antibody and anti-mouse FITC-labelled antibody, counter-stained using hematoxylin-eosin (HE). Cells were viewed under a Zeiss fluorescent microscope for penetration and localization of recombinant proteins.

### Cytotoxic assays

Tumour cells cells (5x10^3^) were seeded in DMEM medium with 10% FCS in the presence of 5% CO_2_ in 96-well plates overnight. Recombinant proteins and/or chemotherapy were added in a concentration gradient in the absence of serum, and incubated for 48 hours. The relevant controls included untreated cells, cells treated with PBS, Antp alone, p21 alone, and cells which were totally lysed with detergent. Cell survival was determined using the Cell Tire-96 kit (Promega). Multiple experiments were performed, and cytotoxicity was expressed as mean percent survival ± SD. Differences between test and control samples were evaluated for statistical significance by t-test.

### Retinoblastoma phosphorylation assays

Cells (5x10^3^) were seeded in 96-well plates, and grown for up to 36 hours. Antp-p21 fusion protein and control media alone was added and incubated for up to 3 hours. The media was removed and the cells grown for a further 3–4 hours. The cells were trypsinised, lysed and the proteins analysed on a 7% acrylamide gel and immunoblotted with anti-retinoblastoma antibodies (Becton Dickinson, USA).

### Studies using animal models

All animal experiments were approved by the Imperial College Centre for Biomedical Sciences Ethical Review Panel and UK Home Office (project licence PPL 70/6982). All procedures and observations complied with UKCCCR (UK co-ordinating committee on cancer research) guidelines.

All animal groups were randomised prior to drug administration and measurements were blinded. Tumour sizes (volume, V) were measured according to the formula V = length x width x width/2. Animals were purchased from Harlan UK Ltd.

### *In vivo* administration of Antp-p21

Female BALB/c athymic mice (6–8 weeks) were implanted with a variety of tumours. Five million cells in 200 μl complete media were subcutaneously implanted into the left flank of mice. The mice were housed in sterilized, individually-ventilated cages. Approximately 21–28 days later when the diameter of tumour was about 0.5 ± 0.2 cm mice were used. Acute mortality and mean maximum body weight loss was used as a measure of relative toxicity.

### Biodistribution and histological studies

For biodistribution analyses, proteins were radiolabelled with Iodine-125 using the IODO-GEN® method (Pierce) and tissues analysed by gamma-counting. All microscopy slides were prepared using standard procedures, stained with anti-pentahistidine antibody and anti-mouse FITC-labelled antibody, counter-stained using HE staining and viewed under a Zeiss fluorescence microscope.

### Treatment studies

Ovarian and colorectal cancer models were used as described in Table 1 (outline of treatment regimens). All doses were administered intravenously and tumours were measured for up to 8 weeks, every other day. For each group, n = 8 animals. An initial experiment was carried out using 8-20 mg/kg Antp vs 8-20 mg/kg Antp-p21 (no chemotherapy). Animals were monitored for mortality and weight loss and were sacrificed when their tumour sizes tripled.

**Table 1:** Summary of treatment schedules and Antp-p21/chemotherapy combinations

**Study No**	**Tumour**	**Group**	**Antp-p21 dose**	**Chemotherapy dose**	**PBS**	**Schedule**
1	Ovarian (SKOV3)	1	20 mg/kg	No	No	1 per week for 5 weeks
1	Ovarian (SKOV3)	2		Cis-Pt[2.5 mg/kg]; Taxol [10 mg/kg]	No	1 per week for 5 weeks
1	Ovarian (SKOV3)	3	20 mg/kg	Cis-Pt[2.5 mg/kg]; Taxol [10 mg/kg]	No	1 per week for 5 weeks
1	Ovarian (SKOV3)	4	No	No	Yes	1 per week for 5 weeks
2	Colon (RKOE6)	1	20 mg/kg	No	No	1 per week for 5 weeks
2	Colon (RKOE6)	2		5FU [2 mg/kg] LV [1 mg/kg] OxPt [0.2 mg/kg]	No	1 per week for 5 weeks
2	Colon (RKOE6)	3	20 mg/kg	5FU [2 mg/kg] LV [1 mg/kg] OxPt [0.2 mg/kg]	No	1 per week for 5 weeks
2	Colon (RKOE6)	4	No	No	Yes	1 per week for 5 weeks
3	Colon (RKOE6)	1	20 mg/kg	No	No	1 per day (Mon-Fri) for 4 weeks
3	Colon (RKOE6)	2		5FU [2 mg/kg] LV [1 mg/kg] OxPt [0.2 mg/kg]	No	Chemo M,W F for 4 weeks
3	Colon (RKOE6)	3	20 mg/kg	5FU [2 mg/kg] LV [1 mg/kg] OxPt [0.2 mg/kg]	No	1 per day (Mon-Fri) for 4 weeks Chemo M,W F
3	Colon (RKOE6)	4	No	No	Yes	1 per day (Mon-Fri) for 4 weeks
4	Colon (Colo205)	1	20 mg/kg	No	No	1 per day (Mon-Fri) for 4 weeks
4	Colon (Colo205)	2		5FU [2 mg/kg] LV [1 mg/kg] OxPt [0.2 mg/kg]	No	Chemo M,W F for 4 weeks
4	Colon (Colo205)	3	20 mg/kg	5FU [2 mg/kg] LV [1 mg/kg] OxPt [0.2 mg/kg]	No	1 per day (Mon-Fri) for 4 weeks Chemo M,W F
4	Colon (Colo205)	4	No	No	Yes	1 per day (Mon-Fri) for 4 weeks

Treatment studies, doses and schedules.

### Immunogenicity of Antp-p21

Four New Zealand White Rabbits were immunized intravenously with 2.5 mg Antp-p21 without adjuvant, once per day for 5 days. Rabbits were bled once per week over a 4-month period. Control rabbits were immunized with saline. Blood samples were diluted 1:10 and 1:100 in saline, and the immune response was monitored by ELISA on native Antp-p21 (coated 50 μg/ml), detected using anti-Rabbit antibodies.

### Statistical analysis

In all experiments, average of all animals were expressed as mean ± SD. Mean tumour burdens in treated animals versus controls were compared at a given time point using the unpaired, two-tailed t-test. Differences between data from two animal groups were assessed by t-test and p < 0.05 was considered significant. Survival was analyzed using the method of Kaplan and Meier and plots were compared for statistical significance using the log-rank test. For tumour growth analysis, the Kruskal-Wallis non-parametric test for multiple comparison analysis was used.

## Results

### Expression and purification of recombinant proteins

**Figure 1:**
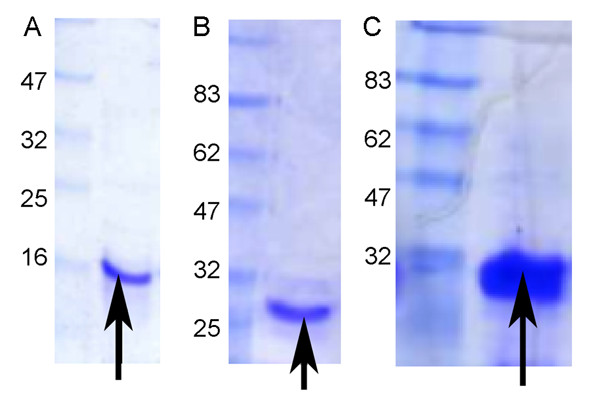
**Coomassie-stained SDS-PAGE of purified recombinant proteins.** (**A**) Antennapedia (Antp) protein (expected molecular weight-11 kDa) (**B**) p21 protein (expected molecular weight-23 kDa) and (**C**) Antp-p21 fusion protein (expected molecular weight-30 kDa) with molecular weight markers. An extra 2–3 kDa is added from the host vector sequence.

Expression of the recombinant proteins resulted in good yields. Antp yielded 20 mg/L of culture, p21, 10 mg/L, whilst Antp-p21 was 5 mg/L after refolding. Expression reduced upon fusing the p21 gene to the Antp gene often seen with chimaeric proteins. Antp-p21 protein precipitation was seen at concentrations higher than 2.5 mg/ml. Figure 1 shows the purified protein analysis by SDS-PAGE which all migrated at their expected molecular weights.

### Intra-nuclear uptake of the Antp-p21 fusion protein

**Figure 2:**
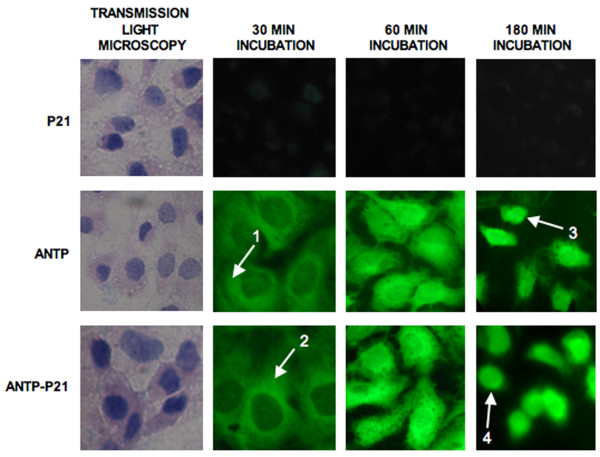
**Intra-nuclear uptake of Antp and Antp-p21 fusion proteins.** Immunofluorescent detection of recombinant proteins administered to cultured cells. On the left, transmission light photographs of SKOV-3 tumour cells incubated with 0.1 mg/ml p21, Antp or Antp-p21 proteins are shown. Trafficking of the proteins is shown in the fluorescent photographs at 30, 60 and 180 min post incubation. Antp and Antp-p21 proteins, contrary to p21, are shown to gradually accumulate in the nucleus of the cells. (1,2) cytoplasm, (3,4) nucleus.

Antp and Antp-p21 were shown to comparably penetrate cancer cells at a 100 μg/ml concentration (representative cells shown in Figure 2) trafficking to the nucleus within 1–3 hours. Cells into which the protein had penetrated intra-nuclearly were scored positive by immunofluorescence. No cellular uptake was seen with free p21 protein alone demonstrating that Antp mediated the cellular delivery.

### *In vitro* inhibition of cellular proliferation by Antp-p21 and mechanism of action

**Figure 3:**
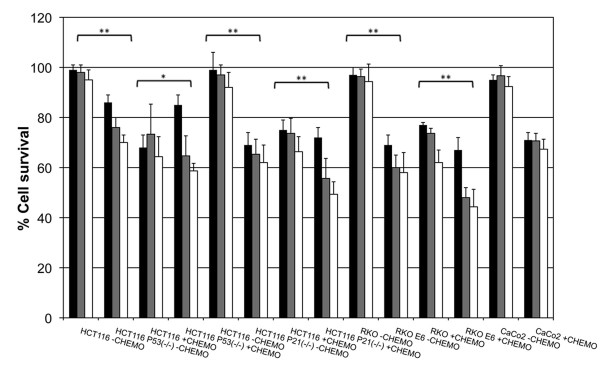
*** In vitro *cellular cytotoxicity on p53/p21-expressing or deficient HCT116 cell lines.** The activity of 5 μg/ml (black bars), 20 μg/ml (grey bars) Antp-p21 fusion protein and chemotherapy alone (white bars) in the absence of chemotherapy or in the presence of 10 μg/ml cisplatin and 8.5 μg/ml paclitaxel was investigated in a series of human colon carcinoma cell lines. The cell lines used were: (**A**) HCT116, p53 parental p21^+^/^+^, p53^+^/^+^. (**B**) HCT116, p53 knock-out p21^+^/^+^, p53^−/−^, (**C**) HCT116, p21 parental p21^+^/^+^, p53^+^/^+^, (**D**) HCT116, p21 knock-out p21^-^/^-^, p53^+^/^+^, (**E**) RKO p53^+^/+, (**F**) RKO E6 (pCMV-E6-transfected RKO)-lacks appreciable functional p53, (**G**) Caco-2 (colon adenocarcinoma line used as control). Bars grouped with a single star (*) are statistically significantly different (=p < 0.05) and bars grouped with a double star (**) are highly statistically significantly different (=p < 0.005) using the student’s T-test.

SKOV-3 cells were incubated with Antp-p21, controls and chemotherapy drugs. Examples of 5 μg/ml and 20 μg/ml in combination with 10 μg/ml cisplatin and/or 8.5 μg/ml paclitaxel are shown (Additional file 1: Figure S1). Antp was not cytotoxic alone and the higher dose of Antp-p21 demonstrated some potency. Moreover, the potency of both doses of Antp-p21 was shown to be enhanced by the chemotherapy. A greater understanding of the mechanism of action for Antp-p21 was revealed upon treatment of genetically-identical human colorectal tumour cell lines (HCT116) that only differ in p53 or p21 gene expression status (Figure 3). Antp-p21 treatment of p53^+^/p21^+^ expressing cell lines at 2 doses (5 μg/ml and 20 μg/ml) did not alter cell survival whereas up to 40% significant inhibition was seen in p53-deficient or p21-deficient HCT116 cells. The effect was more pronounced in the presence of chemotherapy. A similar effect was seen when RKO colorectal cancer cells were compared to E6-virally inhibited p53 (Figure 3).

### Biodistribution, pharmacokinetics and immunogenicity of Antp-p21

**Figure 4:**
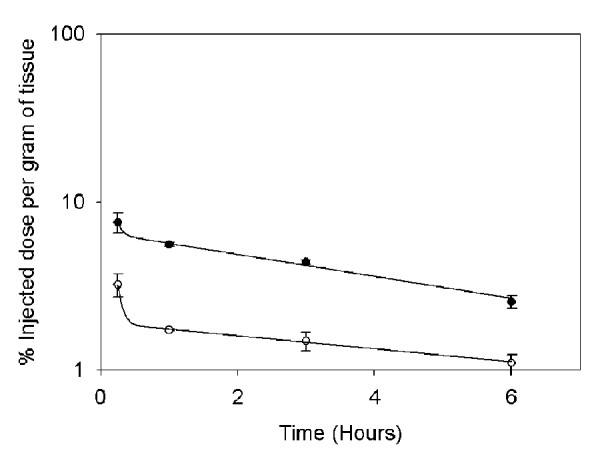
*** In vivo *blood clearance and tumour uptake of the Antp-p21 fusion protein.** Radiolabelled Antp-p21 was injected into SKOV-3 tumour-bearing nude mice and the blood (●) and tumour (○) was counted over 6 hours. The percentage of injected dose per gram of tissue is plotted and fit to a bi-exponential decay curve. For the blood clearance, the decay curve yielded a beta-phase half-life value of 4.6 hours.

Twenty micrograms of radiolabelled Antp-p21 were injected intravenously and all the tissues were dissected/counted over 6 hours. By 3–6 hours, the recombinant protein was distributed across most tissues with no particularly higher uptake in any tissue (Additional file 1: Figure S2). In the early time points, high uptake (8-10% injected dose/g tissue) was seen in the highly-vascularized tissues such as lung, liver and kidney. In general, the tissue distribution reflected the blood clearance which had an elimination clearance rate (t½β) of 4.6 hours (Figure 4). The tumour uptake over the 6 hours is seen to follow that of the blood.

Intravenously-administered Antp-p21 indicated that Antp-p21 does not raise an immune response in immuno-competent rabbits at a dose of 12.5 mg per week (Additional file 1: Figure S3).

### Retinoblastoma phosphorylation

The under-phosphorylated form of Rb is mainly found in resting or fully differentiated cells, whereas the highly phosphorylated forms are present in proliferating cells acting as a marker of cells in G0/G1-phase of the cell cycle. The under-phosphorylated form of Rb is present after Antp-p21 treatment, suggesting that Antp-p21-treated cells lost their ability to phosphorylate newly synthesized Rb at multiple sites and that under-phosphorylated Rb may restrict cell proliferation (Additional file 1: Figure S4).

### Antitumour activity in human tumour xenografts

Details of the dosage and scheduling are listed in Table 1.

### Ovarian adenocarcinoma model

**Figure 5:**
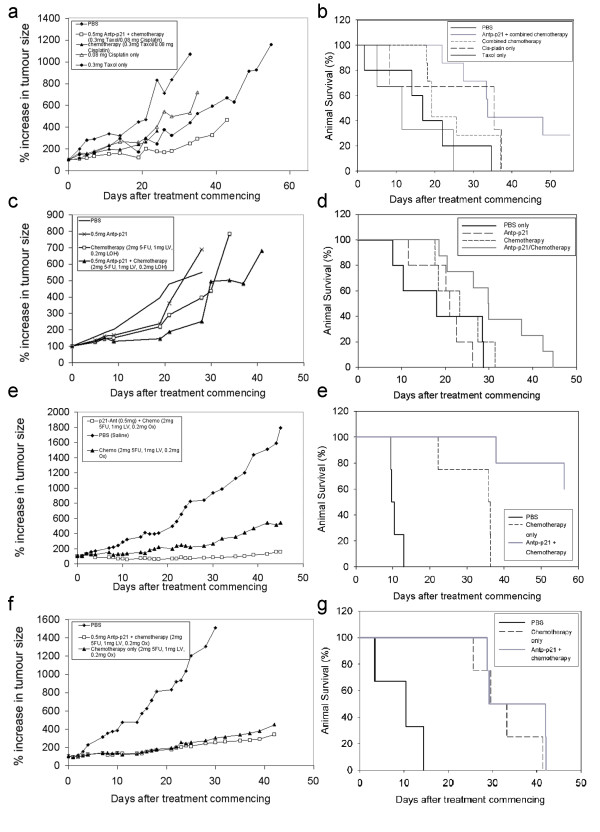
**Combined therapy experiments (see Table**1**for scheduling).** (**A**-**B**) **Single-agent* versus *combination treatment in the SKOV-3 ovarian carcinoma model.** (**A**) Tumour growth analyses. A comparison of the means of the growth curves using multiple comparison analysis demonstrated that 20 mg/kg Antp-p21:2.5 mg/kg cisplatin:10 mg/kg taxol treatment group performed better (smaller tumour burden, lowest mean rank) versus all the other treatment groups (taxol, cisplatin, cisplatin/taxol alone) in a statistically significant manner (*p* = 0.023). The Kruskal-Wallis non-parametric test for multiple comparison analysis demonstrated superiority of the Antp-p21/ cisplatin/taxol treatment group versus all the other treatment groups (*p* = 0.045). (**B**) Kaplan-Meier survival plot. Antp-p21/cisplatin/taxol combination treatment demonstrated a statistically significant increased overall survival versus the other treatment groups (*p* = 0.0204).(**C**-**D**) **Single-agent* versus *combination treatment in the RKO E6 colon carcinoma model.** (**C**) Tumour growth analyses. Treatment with 20 mg/kg Antp-p21:2 mg/kg fluorouracil:1 mg/kg leucovorin: 0.2 mg/kg oxaliplatin combination treatment was superior in terms of tumour growth retardation. (**D**) Kaplan-Meier survival curve. Antp-p21/fluorouracil/oxaliplatin combination treatment was superior in terms of prolonging survival to the other treatment groups (*p* = 0.044) using log rank test and (*p* = 0.018) using independent sample t-test. (**E**-**F**) **Chemotherapy* versus *combination treatment in the RKO E6 colon carcinoma model with a higher dosage schedule.** (**E**) Tumour growth analyses. The Kruskal-Wallis non-parametric test for multiple comparison analysis demonstrated significant reduction on tumour burden in both the treatment groups versus control (20 mg/kg Antp-p21:2 mg/kg 5-fluorouracil: 1 mg/kg leucovorin: 0.2 mg/kg oxaliplatin-treated group *p* = 0.008, 5-fluorouracil/leucovorin/oxaliplatin-treated group *p* = 0.043). (**F**) Kaplan-Meier survival curve. The Antp-p21/5-fluorouracil/oxaliplatin-treated group was superior to that receiving chemotherapy alone with respect to survival (*p* = 0.0027). (**G**-**H**) **Chemotherapy* versus *combination treatment in the Colo 205 colon carcinoma model.** (**G**) Tumour growth analyses. The growth rates of the tumours in these two animal groups (20 mg/kg Antp-p21:2 mg/kg fluorouracil:1 mg/kg leucovorin: 0.2 mg/kg oxaliplatin) vs the same chemotherapy alone demonstrated no significant difference (*p =* 0.773). (**H**) Kaplan-Meier survival curve. The animal group receiving chemotherapy demonstrated a median overall survival of 29.76 days whereas the group receiving both chemotherapy and fusion protein 29.33 days (*p =* 0.349).

The anti-tumour activity of increasing doses injected over 5 weeks of Antp-p21 was compared to that of controls in the SKOV-3 ovarian carcinoma xenograft model. Minor but observable growth delays and increased survival was demonstrated (Additional file 1: Figure S5). Better effects were seen when Antp-p21 was combined with conventional ovarian cancer chemotherapy. Antp-p21 (0.2 ml of 2.5 mg/ml equalling 20 mg/kg) was administered once a week for five weeks in combination with 2.5 mg/kg cisplatin and 10 mg/kg taxol with appropriate controls. Figure 5a shows that Antp-p21, cisplatin, taxol, and the combination of taxol/cisplatin were only modestly active against this tumour, whilst the combination of Antp-p21/cisplatin/taxol was significantly more effective, with significant growth delay (p = 0.023). Little toxicity was observed for all the treatment regimens. However, on cessation of treatment, the tumours grew at approximately the same rate. The Kruskal-Wallis non-parametric test for multiple comparison analysis demonstrated superiority of the Antp-p21/cisplatin/taxol treatment group versus all the other treatment groups (p = 0.045). This group demonstrated a statistically significant (p = 0.0204) increased overall survival compared to the other treatment groups, (Figure 5b).

### Colorectal cancer model

Antp-p21 was investigated in the RKOE6 model. The regimen was the same as described for the ovarian cancer model (Table 1), however, the supplementary chemotherapy was modified into one for colorectal carcinomas (modified De Gramond protocol [29]-combination of 2 mg/kg fluorouracil/ 1 mg/kg leucovorin with 0.2 mg/kg oxaliplatin). This is considered as the standard therapy currently used for metastatic colon cancer. Figure 5c demonstrates the enhanced tumour growth inhibitory activity of 20 mg/kg Antp-p21 when used in combination with this protocol. Tumours in the Antp-p21/fluorouracil/leucovorin/oxaliplatin group experienced near-complete regression during therapy in contrast to those in the other treatment groups. This combination treatment was superior in terms of prolonging survival (Figure 5d) versus the other treatment groups (p = 0.044) using log rank test and (p = 0.018) using independent sample t-test.

In this model, the therapy was expanded to a faster dosing schedule: the same chemotherapy was administered three times per week for four weeks with/without the 20 mg/kg Antp-p21 fusion protein (once per day for 5 days for four weeks). Beneficial effects were seen when this regimen was compared to the results of the previous study, suggesting a dose response effect of the total fusion protein delivered to the tumour (Figure 5e). No increased apparent toxicity was seen with increased dose administration; animal weights, behaviour and appearance were similar in all groups. Superior efficacy (Figure 5e) and improved survival (Figure 5f) were demonstrated by the animal group receiving Antp-p21 in combination with chemotherapy compared to chemotherapy alone. For the growth curve (Figure 5e), the Kruskal-Wallis non-parametric test for multiple comparison analysis demonstrated significant reduction in tumour burden in both the treatment groups versus control (Antp-p21/5-fluorouracil/oxaliplatin-treated group p = 0.008, 5-fluorouracil/oxaliplatin-treated group p = 0.043). The Antp-p21/5-fluorouracil/oxaliplatin-treated group was superior to that receiving chemotherapy alone with respect to survival (p = 0.0027), demonstrating statistical significance (Figure 5f). Complete tumour regression rate in the Antp-p21/5-fluorouracil/oxaliplatin-treated group was 40% and response-to-treatment rate was 100%, whilst in the chemotherapy-receiving group these were 0% and 100% respectively. Both groups were significantly superior to the PBS control group (p < 0.001).

Finally, the p21-wild type Colo-205 [30] model was as a negative tumour control with the same rapid dosing as above. In our hands, the model was more reliable to establish *in vivo* and behaves similarly to the RKO cells *in vitro* (data not shown). The growth rates of the tumours in these two animal groups demonstrated no significant difference (p = 0.773) (Figure 5g) with no toxicity seen with increased dose administration. For animal survival (Figure 5h), the animal group receiving chemotherapy demonstrated a median overall survival of 29.76 days whereas the group receiving both chemotherapy and fusion protein 29.33 days (p = 0.349). Both treatments were superior to PBS (p = 0.0012).

## Discussion

In cells where p53 or p21 function is defective, responses to genotoxic stress are also impaired. This frequently leads to resistance to chemotherapeutic drugs. Examples of such genotoxic exposures include cisplatin, which forms DNA adducts that cannot be repaired by the excision repair system [31], oxaliplatin, which forms reactive platinum complexes with DNA, thus inhibiting synthesis, paclitaxel, which disrupts mitosis by interacting with the assembly of microtubules, and fluorouracil which results in decreased DNA synthesis and repair, and ultimately decreased cell proliferation. Consequently, restoration of wild-type p53 or p21 functions is seen as a particularly promising approach for cancer therapy.

We have developed a fusion protein, Antp-p21 comprising of a transduction domain antennapedia and a cyclin-dependent kinase inhibitor p21 that penetrates indiscriminately living cells including penetrating through the blood brain barrier. In all animal experiments, no demonstrable toxicity or immunogenicity were seen, but these were very much preliminary observations. Further work in more elaborate animal models would be needed. Antp-p21 was found to penetrate *in vitro* and *in vivo* and kill cancer cells derived from different tissues. Non-specific toxicity of Antp-p21 was excluded by the lack of cytotoxicity for Colo-205 cells, unresponsive to p21. On the other hand the degree of cytotoxicity of Antp-p21 for cancer cells with absent expression of p53 or p21 was remarkable.

Our proposed mechanism of action is that the delivery of functional p21 is able to restore p21 function in tumours resulting in tumour apoptosis and regression upon exposure to chemotherapy in two human tumour models. This mechanism is supported by the fact that p53 or p21 wild-type tumour cells failed to respond to this combination therapy. Our approach is further supported by the use of cogenic human tumour cells lines, which only differ in p53 and p21 status. This system eliminates the unpredictability due to other undefined genetic mutations. We observed that HCT116 cells deficient in p21 (directly or via p53 control) were more sensitive to Antp-p21 therapy compared to parental cell line controls. The effect was also seen in RKO cells. The difference was clear and significant and supports our *in vivo* observations.

Therapeutic efficacy was related to the frequency and intervals between agent administrations; treatment with Antp-p21 simultaneously with chemotherapy every 72 hours was significantly superior to therapy given on a weekly basis. The Antp-p21 fusion protein has a fast clearance rate *in vivo*, likely due to its small molecular weight. This may also explain the dose scheduling observations. Although detailed pharmacokinetic studies have not been carried out in mice, it is likely that the optimal administration of Antp-p21 would be in conjunction with conventional chemotherapy, possibly within a stabilizing formulation [32].

As previously documented in the metastatic setting of colorectal cancer [33], greater p21 presence in tumours resulted in greater chemosensitivity. Our results further support this view in the non-metastatic setting. p21 has been previously shown to be the mediator of cell cycle phenomena in colorectal carcinoma cells and to play a critical role in apoptotic responses [34]. In the present study, we speculate that the mechanism of action is the induction of cellular senescence and apoptosis after exposure to genotoxic insults. This mechanism is achieved due to the restoration of the previously-corrupted p21 function and other downstream pathways of the cell cycle. The microscopy data and the retinoblatoma status data support this view, but a more detailed mechanistic analysis is in progress.

## Conclusions

This study proposes a new paradigm, where based on genomic selection of tumour types, the fusion protein Antp-p21 can act on its own and also enhance the efficacy of chemotherapy leading to tumour eradication in p53- or p21-mutated tumours. This work supports the concept of developing individualised therapy for cancer based on the mutation status of patients’ cancers but needs, of course, to be demonstrated in a clinical setting.

## Competing interests

Dr Kousparou and Professor Epenetos are co-founders of Trojantec who are developing the antennapedia platform. Dr Kousparou and Professor Epenetos also own equity in the form of shares in Trojantec Ltd.

## Authors’ contributions

CK and AAE conceived the study ideas. CK and EY carried out the *in vitro* cell-based work, MPD and CK performed the *in vivo* animal model experiments, CK, MPD nad AAE analysed and interpreted the data and all four authors prepared, wrote, read and approved the manuscript.

## Pre-publication history

The pre-publication history for this paper can be accessed here:

http://www.biomedcentral.com/1471-2407/12/338/prepub

## Supplementary Material

Additional file 1 **Figure S1. In vitro potency of Antp-p21.** Antp-p21 at 5μg/ml (black bars) and 20μg/ml (grey bars) was incubated with SKOV3 (which are p53 deficient) with various combination of chemotherapy drugs (Cisplatin (10 μg/ml), and Taxol (8.54 μg/ml). The double combination together with Antp-p21 showed an additive effect in reducing cell proliferation. **Figure S2. Biodistribution of Antp-p21 in tumour-bearing mice.** Radiolabelled Antp-p21 was injected into tumour-bearing mice and tissues analysed by gamma counting over 15 minutes to 18 hours. A high level of Antp-p21 was observed in the majority of the major organs which cleared after around 1 hour. No net accumulation was seen for any particular tissue. **Figure S3. Immunogenicity of Antp-p21 in rabbits.** Antp-p21 did not raise an immune response in immuno-competent rabbits at a dose of 12.5 mg over a week of immunizations. **Figure S4. Rb-phosphorylation assay of Antp-p21 treated cells.** Antp-p21 (20μg/ml) was shown to Inhibit the phosphorylation of retinoblastoma protein (Rb) in SKOV3 cells compared to a non-treated control. **Figure S5. Efficacy of Antp* versus *Antp-p21 treatment – Dose escalation study.** Mice (8/group) with s.c. SKOV-3 tumours received either Antp alone in increasing dosages (once per week for five weeks), or Antp-p21 alone in increasing dosages (once per week for five weeks), or PBS (once per week for five weeks). (A) Tumour growth during study after the first i.v. administration. (B) Kaplan-Meier survival of animals following these treatments. Click here for file
