# Supplementary material for: Generation of a selectively cytotoxic fusion protein against p53 mutated cancers
Source: BMC Cancer. 2012 Aug 3;12:338. doi: 10.1186/1471-2407-12-338 (PMC3503689; doi:10.1186/1471-2407-12-338)
Supplement: Additional file 1 — Figure S1. In vitro potency of Antp-p21. Antp-p21 at 5μg/ml (black bars) and 20μg/ml (grey bars) was incubated with SKOV3 (which are p53 deficient) with various combination of chemotherapy drugs (Cisplatin (10 μg/ml), and Taxol (8.54 μg/ml). The double combination together with Antp-p21 showed an additive effect in reducing cell proliferation. Figure S2. Biodistribution of Antp-p21 in tumour-bearing mice. Radiolabelled Antp-p21 was injected into tumour-bearing mice and tissues analysed by gamma counting over 15 minutes to 18 hours. A high level of Antp-p21 was observed in the majority of the major organs which cleared after around 1 hour. No net accumulation was seen for any particular tissue. Figure S3. Immunogenicity of Antp-p21 in rabbits. Antp-p21 did not raise an immune response in immuno-competent rabbits at a dose of 12.5 mg over a week of immunizations. Figure S4. Rb-phosphorylation assay of Antp-p21 treated cells. Antp-p21 (20μg/ml) was shown to Inhibit the phosphorylation of retinoblastoma protein (Rb) in SKOV3 cells compared to a non-treated control. Figure S5. Efficacy of Antp versus Antp-p21 treatment – Dose escalation study. Mice (8/group) with s.c. SKOV-3 tumours received either Antp alone in increasing dosages (once per week for five weeks), or Antp-p21 alone in increasing dosages (once per week for five weeks), or PBS (once per week for five weeks). (A) Tumour growth during study after the first i.v. administration. (B) Kaplan-Meier survival of animals following these treatments. [file 1471-2407-12-338-S1.docx]

**Supplementary Figures**

**Supplementary Figure 1. In vitro potency of Antp-p21.**

Antp-p21 at 5μg/ml (black bars) and 20μg/ml (grey bars) was incubated with SKOV3 (which are p53 deficient) with various combination of chemotherapy drugs (Cisplatin (10 μg/ml), and Taxol (8.54 μg/ml). The double combination together with Antp-p21 showed an additive effect in reducing cell proliferation.

**Supplementary Figure 2. Biodistribution of Antp-p21 in tumour-bearing mice**

Radiolabelled Antp-p21 was injected into tumour-bearing mice and tissues analysed by gamma counting over 15 minutes to 18 hours. A high level of Antp-p21 was observed in the majority of the major organs which cleared after around 1 hour. No net accumulation was seen for any particular tissue.

**Supplementary Figure 3. Immunogenicity of Antp-p21 in rabbits**

Antp-p21 did not raise an immune response in immuno-competent rabbits at a dose of 12.5 mg over a week of immunizations.

**Supplementary Figure 4. Rb-phosphorylation assay of Antp-p21 treated cells**

Antp-p21 (20μg/ml) was shown to Inhibit the phosphorylation of retinoblastoma protein (Rb) in SKOV3 cells compared to a non-treated control.

**Supplementary Figure 5. Efficacy of Antp *versus* Antp-p21 treatment – Dose escalation study**

Mice (8/group) with s.c. SKOV-3 tumours received either Antp alone in increasing dosages (once per week for five weeks), or Antp-p21 alone in increasing dosages (once per week for five weeks), or PBS (once per week for five weeks). (A) Tumour growth during study after the first i.v. administration. (B) Kaplan-Meier survival of animals following these treatments.
